# Toward a neuroergonomic understanding of spatial disorientation

**DOI:** 10.3389/fphys.2026.1912219

**Published:** 2026-08-12

**Authors:** Jerome Carriot, Isabelle Mackrous, Pascale Brochu, Simon Corcos, Marc-Antoine Pelletier, Maurice J. Chacron

**Affiliations:** 1 Department of Physiology, McGill University, Montreal, QC, Canada; 2 HOP Technologies, Sherbrooke, Bromont, Montreal, QC, Canada

**Keywords:** motion perception, neuroergonomics, spatial disorientation, vestibular illusion, vestibular system

## Abstract

Despite decades of research, spatial disorientation, which is the incorrect perception of one’s orientation relative to gravity, remains one of the leading causes of aviation mishaps. In this mini-review, we highlight the role of the vestibular system towards generating disorientation. Specifically, in the absence of other cues, the brain is incapable of distinguishing between the acceleration caused by a static force such as gravity from that experienced in an accelerating vehicle, which leads to an ambiguity leading to acceleration being wrongly interpreted as tilts. Finally, because vestibular signals interact with autonomic, respiratory, and vascular regulation, we propose multimodal physiological monitoring as a testable research strategy for characterizing unstable self-motion estimation, while emphasizing that percept-specific detection requires controlled labeling and prospective validation.

## 1 Introduction

Fast-jet and next-generation aviation place pilots in conditions where sustained acceleration, degraded external visual references, and high cognitive demand can destabilize the perception of motion, attitude, and gravity (Demir and Aydin, 2021). Spatial disorientation remains dangerous in this context because pilots may act on a false but internally coherent estimate of aircraft state. This risk reflects a fundamental constraint of vestibular processing: otolith signals encode gravito-inertial acceleration and therefore cannot, by themselves, distinguish head tilt from linear acceleration (Mach, 1875; Fernandez et al., 1972; Paige and Tomko, 1991). When visual references are degraded and canal cues are limited or misleading, central internal models can resolve this tilt-translation ambiguity incorrectly, producing compelling illusions such as the somatogravic illusion. Because vestibular processing is coupled to autonomic, respiratory, and vascular regulation, multimodal neuroergonomic and homeodynamic monitoring may help detect unstable self-motion estimation and support individualized adaptive countermeasures. These points are discussed in the following paragraphs, from the operational persistence of spatial disorientation to its vestibular mechanisms and potential physiological markers.

## 2 Spatial disorientation: a persistent systems-level failure

Spatial disorientation remains one of the most consequential human-factor limitations in aviation. It is commonly defined as the inability to correctly perceive orientation, position, or motion relative to the Earth, and it can lead to inappropriate control inputs despite intact sensory organs and otherwise normal cognitive function (Demir and Aydin, 2021). This distinction is essential: spatial disorientation is not simply a lapse in attention, discipline, or instrument knowledge. It reflects a failure of the integrated human-aircraft system to maintain an accurate estimate of orientation under conditions that exceed the assumptions of everyday self-motion.

The operational burden of spatial disorientation has persisted for decades. Gibb et al. (2011) emphasized that spatial disorientation continues to contribute disproportionately to aviation fatalities, while Poisson and Miller (2014) showed that it often appears embedded within broader accident sequences rather than as an isolated causal label. This under-recognition matters because spatial disorientation may be hidden behind outcomes such as loss of control, controlled flight into terrain, task saturation, or poor instrument cross-check. In many cases, the final control input is visible, but the upstream failure is an internal orientation estimate that has already diverged from aircraft reality.

This problem is likely to become more, no less, relevant in next-generation aviation. Fifth- and sixth-generation fighter operations combine rapid acceleration transitions, high-G maneuvering, helmet-mounted displays, fused sensor products, degraded external visual references, and intense cognitive demands. These technologies can improve situation awareness, but they also increase the density of information that must be interpreted under time pressure. Thus, the modern pilot may have unprecedented access to synthetic information while simultaneously experiencing compelling biological signals that misrepresent motion and orientation.

**Figure 1:**
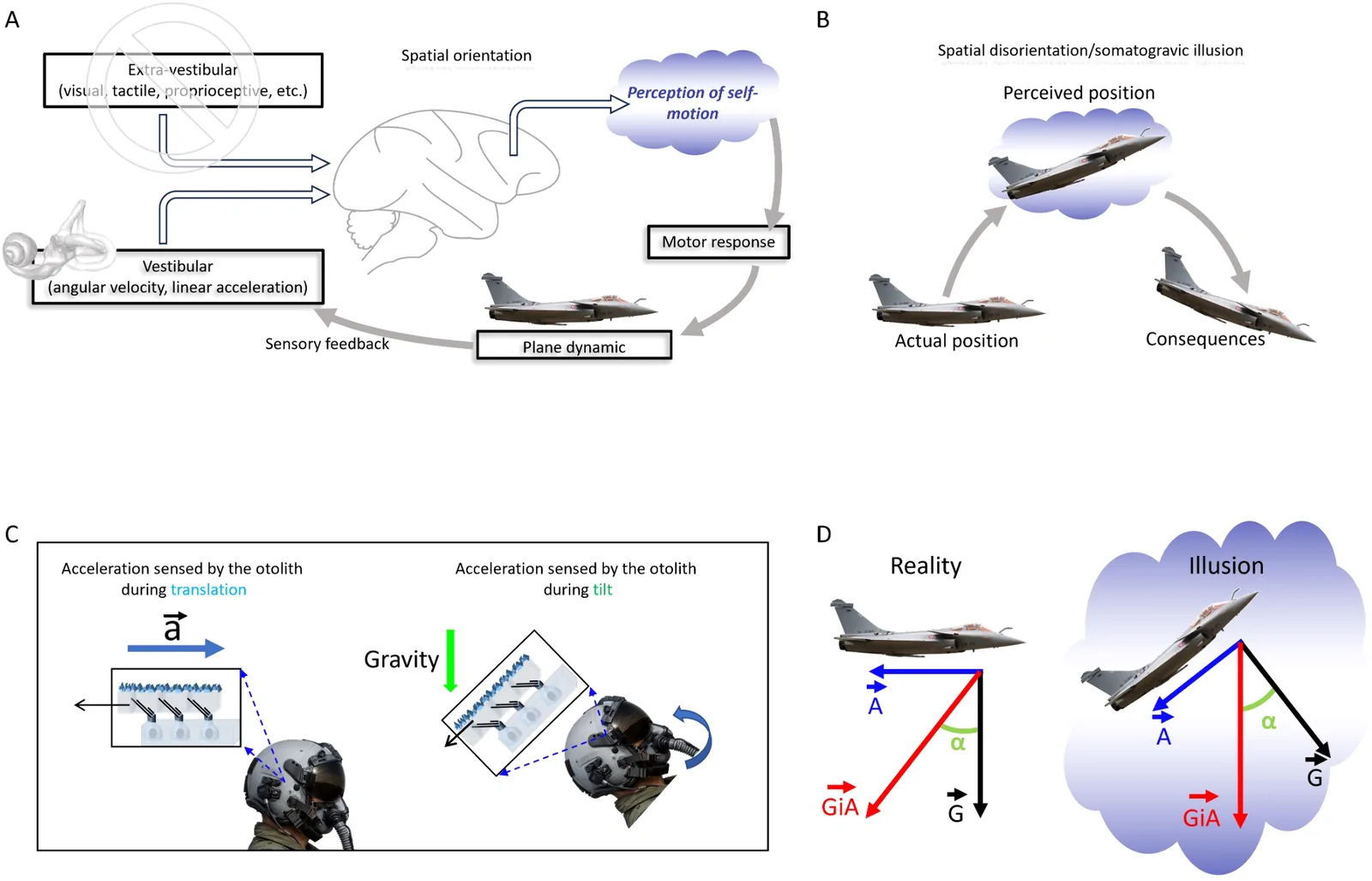
Vestibular basis of spatial disorientation and the somatogravic illusion. **(A)** Spatial orientation normally depends on the brain integrating vestibular, visual, proprioceptive, somatosensory, and motor-related cues to estimate self-motion and orientation relative to gravity. When external visual and non-vestibular cues are degraded or unreliable, as can occur in fast-jet flight, the central estimate of self-motion must rely more heavily on vestibular signals that are informative but ambiguous. **(B)** Spatial disorientation can arise when the aircraft’s actual attitude differs from the pilot’s perceived attitude. During sustained forward acceleration, the pilot may perceive the aircraft as pitched upward even when the aircraft is not actually in that attitude. This false but compelling perceived position can lead to inappropriate corrective control inputs, shown here as a potential nose-down consequence. **(C)** Otolith organs sense gravito-inertial acceleration rather than gravity and linear acceleration as separate signals. During translation, inertial acceleration deflects the otolithic sensor in a way that can resemble the otolith response produced by head tilt relative to gravity. During tilt, gravity produces a comparable shear force across the otolith epithelium. Because these two physical situations can generate similar otolith activation patterns, otolith input alone is ambiguous with respect to translation versus tilt. **(D)** In real forward acceleration, the aircraft remains level while the resultant gravito-inertial acceleration vector is shifted by the combination of gravity (G) and inertial acceleration **(A)**. Without reliable visual or other orientation cues, the brain may interpret this shifted gravito-inertial vector (GIA) as evidence that the aircraft is tilted nose-up by an apparent angle (α). This perceptual reinterpretation is the somatogravic illusion: the sensed force vector is real, but the inferred aircraft attitude is incorrect.

The persistence of spatial disorientation arises from the way the central nervous system estimates self-motion. Under natural terrestrial conditions, spatial orientation is an active multisensory inference: the brain combines visual, vestibular, proprioceptive, somatosensory, and motor efference-related cues into a stable estimate of body and head orientation (**Figure 1A**). Vision usually anchors this estimate through the horizon, optic flow, and allocentric landmarks. In flight, however, these visual constraints may be degraded or attentionally inaccessible, forcing greater reliance on vestibular and somatosensory cues that are informative but inherently ambiguous (Demir and Aydin, 2021a; **Figure 1A**).

Spatial disorientation is particularly dangerous not merely because of uncertainty; but also, by generating coherent but false percepts. The pilot may feel a convincing sense of pitch, roll, climb, descent, or acceleration that conflicts with the actual aircraft state. The somatogravic illusion is a critical example. During forward acceleration, such as takeoff, catapult launch, afterburner engagement, or go-around, the resultant gravito-inertial vector shifts backward relative to the head. In the absence of reliable visual cues, this can be perceived as backward tilt or an excessive nose-up attitude, leading the pilot to pitch the aircraft downward (Clark and Graybiel, 1949; DiZio et al., 1997; Carriot et al., 2005; Carriot et al., 2006; Carriot et al., 2008; **Figure 1B**). The resulting control input can further increase acceleration and reinforce the very illusion that triggered it.

Experimental evidence confirms that this is not merely a theoretical hazard. Somatogravic stimulation alters perceived altitude and can degrade flight performance, including by reducing climb rate or promoting inappropriate pitch control (Socha et al., 2026). These effects also vary substantially across individuals (Carriot et al., 2006), indicating that the same acceleration profile can produce different perceptual and behavioral outcomes across pilots. Importantly, training does not eliminate this vulnerability: Socha et al. (2026) reported that pilots may improve performance across repeated simulator exposures while still failing to explicitly recognize or report the illusion. Thus, behavioral adaptation, conscious awareness, and perceptual reliability can dissociate.

This dissociation explains why traditional mitigation remains incomplete. Classroom instruction can teach pilots to recognize named illusions, and simulators can expose them to selected risk scenarios, but neither approach directly reveals the internal state in which perception becomes unreliable. The most dangerous cases may be those in which the false percept is accepted as reality and never becomes available for explicit correction. Addressing spatial disorientation therefore requires moving beyond description and training toward mechanism: when self-motion estimation fails, why it fails, and in whom it is most likely to fail.

## 3 Tilt-translation ambiguity: a fundamental computational constraint

The persistence of spatial disorientation can be traced to a fundamental constraint imposed by physics and neural encoding: the tilt-translation ambiguity that emerges from the biomechanics of vestibular sensors. Specifically, the vestibular sensors include the semicircular canals that detect head rotation and the otolith organs that detect the gravito-inertial acceleration (GIA) which is the vector sum of gravitational acceleration and inertial acceleration due to translation (**Figures 1C, D**). Because gravity and sustained linear acceleration are physically indistinguishable at the level of the otoliths, otolith afferents cannot by themselves determine whether a change in the resultant force vector reflects head tilt relative to gravity, linear translation, or some combination of both (Mach, 1875; Fernandez et al., 1972; Paige and Tomko, 1991). This is the aviation-relevant expression of Einstein’s equivalence principle (**Figure 1D**). While the otolith signal is informative, it nonetheless carries ambiguous information about head tilt and translation.

However, this ambiguous signal is sent to the otolith pathway that appears functionally heterogeneous. Based on their spontaneous firing regularity, Jamali et al. (2019) showed that regular and irregular otolith afferents use different coding strategies: irregular afferents transmit more information about dynamic translational motion through temporally precise and nonlinear responses, whereas regular afferents better discriminate static orientation relative to gravity. The frequency segregation theory (Mayne, 1974; Telford et al., 1997; Merfeld et al., 2005; Park et al., 2006) suggests that tilt-translation ambiguity can be resolved at the periphery along the otolith’s pathways. This division may be useful under natural conditions, where motion tends to contain coupled dynamic and postural components (Carriot et al., 2014; Carriot et al., 2017a; Carriot et al., 2017b). In fast-jet aviation, however, the same division may become unreliable because prolonged or low-frequency acceleration can resemble the statistics of tilt, while abrupt maneuvers may exceed the range of everyday self-motion for which these codes are calibrated. On the other hand, but not mutually exclusive, the dominant theoretical framework proposes that resolving tilt-translation ambiguity occurs through central computation. The nervous system constructs an internal estimate of gravity by combining otolith inputs with rotational information from the semicircular canals (Angelaki et al., 1999; Mergner and Glasauer, 1999; McIntyre et al., 2000; Merfeld et al., 2001; Green et al., 2004; Zupan et al., 2004; Green and Angelaki, 2010; Laurens and Angelaki, 2020). Canal signals specify angular velocity and can be integrated to estimate changes in head orientation over time. When canal-derived orientation change is consistent with the otolith signal, the brain can attribute part of the GIA to gravity. When the otolith signal changes without corresponding canal evidence for tilt, the brain can attribute more of the signal to translation. In practice, this computation requires an internal model (McIntyre et al., 2000; Merfeld et al., 2001; Green et al., 2004; Zupan et al., 2004; Green and Angelaki, 2010; Mackrous et al., 2019; Laurens and Angelaki, 2020) that incorporates sensory dynamics, prior expectations about natural motion, and assumptions about how gravity behaves. This framework is powerful because it explains both normal perception and illusion. During everyday motion, the brain can usually infer that the body is tilting, translating, or both by integrating vestibular inputs from the semicircular canals and otoliths with other modalities such as vision, proprioception, and motor predictions, to give rise to accurate perception of orientation. During sustained aircraft acceleration, however, the semicircular canal responses decay after the initial transient, whereas otolith afferents continue to encode the shifted gravito-inertial acceleration (GIA) vector. As canal signals fade, estimates of orientation must increasingly rely on otolith input, prior expectations, and any remaining visual or somatosensory constraints. In sensory-impoverished environments, as these additional cues become unreliable or absent, the internal model is forced to resolve the inherent ambiguity of otolith signals with reduced influence from other sensory inputs.

Conventional models conceptualize self-motion perception as a form of probabilistic inference in which the central nervous system continuously combines weighted sensory evidence with prior expectations about natural motion and gravity (Kording and Wolpert, 2006; Laurens and Droulez, 2007; MacNeilage et al., 2007). A useful way to express this is that the brain does not treat every cue as equally trustworthy at every moment; it weights cues according to their expected precision and consistency. Under typical conditions, vision can rapidly dominate when a stable horizon or coherent optic flow is available, but these cues can become unreliable or unavailable in darkness, cloud, or synthetic display environments. Under these conditions, the vestibular system becomes the dominant sensory source despite conveying inherently ambiguous information. How, then, does the brain construct percepts from vestibular signals? One possibility is that perception emerges through inference, whereby incoming sensory signals are interpreted relative to prior expectations about self-motion and environmental statistics. Because natural environments rarely expose humans to prolonged, visually unreferenced horizontal accelerations (Carriot et al., 2014), the brain may preferentially interpret the resultant gravito-inertial signal as reflecting gravity, and therefore head or aircraft tilt rather than translation (Mach, 1875; Howard and Templeton, 1963; Clark and Graybiel, 1968; Carriot et al., 2005; Carriot et al., 2006). Within this framework, vestibular illusions arise during sustained acceleration profile when the central computation has fewer sensory cues with which to reject the tilt interpretation, causing the inference to yield a virtual tilt percept—an orientation estimate that is internally coherent yet externally incorrect.

Neurophysiological studies provide direct support for this computational view. Laurens et al. (2013) showed that neurons in vestibulocerebellar circuits can encode tilt and translation separately, with population activity reconstructing the full GIA signal. These findings indicate that the brain does not passively read out the otolith organs; it transforms ambiguous peripheral input into estimated physical variables. Mackrous et al. (2019) further demonstrated that cerebellar circuits generate predictions of the sensory consequences of gravity, suppressing expected inputs during active motion while preserving sensitivity to unexpected perturbations. In this sense, the cerebellum and vestibular nuclei are not merely relays but predictive estimators of self-motion.

The most revealing evidence comes from conditions in which canal information is unavailable or uninformative. Laurens et al. (2013) found that when canal cues are absent, vestibular neurons that selectively encode tilt motion progressively lose their ability to encode gravity and become more similar to neurons that selectively encode translation motion. This neural reorganization suggests that tilt and translation selectivity are not hardwired; they emerge from an ongoing computation that depends on the availability and reliability of multisensory evidence. However, this neural reorganization reveals a striking paradox: under these same conditions—such as during somatogravic stimulation—the perceptual experience evolves in the opposite direction, shifting from an initial sensation of translation toward a compelling illusion of tilt.

These findings collectively indicate that self-motion perception is not a direct readout of sensory input but rather the result of a distributed, nonlinear computation involving multiple stages of processing. Importantly, this computation is tuned to the statistics of natural motion (Carriot et al., 2014; Schneider et al., 2015; Carriot et al., 2017a; Carriot et al., 2017b; Mitchell et al., 2018; Mackrous et al., 2020; Carriot et al., 2022; Mohammadi et al., 2024; Carriot et al., 2026). When motion dynamics fall outside this natural range—such as during sustained aircraft acceleration—the internal model can fail. Under these conditions, the brain defaults to interpreting GIA as tilt, resulting in the emergence of a “virtual tilt” percept that does not correspond to physical reality. Thus, vestibular illusions are not simply errors but represent predictable consequences of an otherwise optimal computational strategy operating under extreme conditions. The somatogravic illusion, in particular, can be understood as a direct manifestation of the tilt–translation ambiguity when the internal model fails to correctly estimate gravity.

This interpretation also clarifies why individual differences are expected rather than incidental (Carriot et al., 2005). Pilots may differ in peripheral vestibular dynamics, reliance on visual versus vestibular cues, prior exposure to unusual acceleration profiles, susceptibility to motion sickness, and the speed with which they reweigh conflicting information. This variability should be treated as a continuum rather than as a binary distinction between normal and abnormal spatial orientation. Even among healthy adults, vestibular perceptual thresholds vary substantially across individuals and increase with age (Strupp et al., 2017; Strupp et al., 2023). A mechanistic model of spatial disorientation must therefore account for both the shared physics of tilt-translation ambiguity and the pilot-specific parameters that shape its behavioral expression. For next-generation aviation, the implication is clear: countermeasures must do more than present better external information; they must detect when the pilot’s internal estimate of gravity is becoming unstable and support its recalibration in real time.

## 4 Vestibulo-autonomic coupling: a testable path toward physiological state estimation

Sections 2 and 3 identify spatial disorientation as a failure of self-motion estimation rather than a simple lapse in attention or training. The operational difficulty is that a false percept can become compelling before the pilot recognizes a conflict and may never be explicitly reported (Socha et al., 2026). Although psychophysical tasks can quantify vestibular illusions in the laboratory, they cannot be continuously imposed during flight. Physiological state estimation therefore offers a possible complementary approach. The hypothesis, however, must be stated precisely: autonomic and neural signals may contain information about the pilot’s gravito-inertial stimulation and sensory conflict, but they have not yet been shown to uniquely encode the conscious percept of tilt.

The physiological basis for this hypothesis is the vestibular contribution to feedforward cardiovascular and respiratory regulation. During postural reorientation, the reduction in central blood volume and ventricular preload lowers stroke volume through the Frank-Starling relationship. Baroreflex mechanisms respond to the resulting hemodynamic disturbance, whereas vestibulosympathetic pathways can be recruited at movement onset, before blood redistribution or a fall in arterial pressure has fully developed. Otolith-related signals reach autonomic circuits through the caudal vestibular nuclei, lateral medullary reticular formation, and rostral ventrolateral medulla, where they interact with baroreceptor and cardiopulmonary inputs (Doba and Reis, 1974; Yates, 1992; Yates and Miller, 1994; Woodring et al., 1997; Yates and Miller, 1998; Yates et al., 2014). In humans, brief backward head drops accelerate heart rate within approximately 500 ms in participants with intact vestibular function, but not in patients with vestibular deficits (Radtke et al., 2000). Vestibular stimulation can also modify respiratory frequency and cardiovascular variability (Jauregui-Renaud et al., 2000; Jauregui-Renaud et al., 2001). These findings establish rapid vestibulo-autonomic coupling; they do not, by themselves, establish a physiological marker of an illusion.

This distinction is particularly important for the somatogravic illusion. Because the otolith organs cannot separate tilt from translation, this physically real but ambiguous input can engage brainstem autonomic pathways regardless of how it is ultimately interpreted by perceptual circuits. An autonomic response therefore does not require a top-down command that reproduces the physiology of a nonexistent tilt. In fact, galvanic vestibular stimulation has been shown to modulate heart rate, blood pressure, muscle sympathetic nerve activity, and skin sympathetic nerve activity without reorienting the body or producing an orthostatic fluid shift, although effects vary substantially with stimulation parameters (Pliego and Soto, 2025). During parabolic flight, masking vestibular input with galvanic stimulation abolished the initial arterial-pressure responses to transitions into hypergravity and microgravity (Iwata et al., 2011). However, galvanic stimulation activates canal and otolith afferents in parallel, with dynamics that differ from natural motion, and produces a population activation pattern with no direct physiological equivalent (Kwan et al., 2019). Thus, while galvanic stimulation constitutes a useful mechanistic perturbation, it is not a quantitative surrogate for the somatogravic illusion.

Evidence that physiological activity accompanies illusory self-motion is suggestive but heterogeneous. Visually induced tilt or vection can produce cardiovascular responses in some stationary participants, yet the direction and amplitude vary markedly across individuals and group-level effects are often weak (Aoki et al., 2000; Wood et al., 2000). When visual flow and physical vestibular stimulation were combined, visual stimulation modulated heart rate but did not change mean arterial pressure, and its effect was overridden by vestibular stimulation (Kuldavletova et al., 2023). More directly, an experimentally induced pitch illusion increased heart rate, heart-rate variability, and mean arterial pressure while degrading flight performance in a simulator (Cheung et al., 2004). Wrist-worn electrodermal activity recorded during spatial-disorientation training covaried with marked autonomic symptoms in 177 pilot candidates (Tamura et al., 2018). A recent simulation study in five instrument-rated pilots likewise reported changes in electroencephalographic, cardiac, and respiratory signals during disorientation, while performance alone was insufficient to identify the state (Geva et al., 2025). Together, these studies show that multimodal physiological measurements can capture state changes during vestibular illusions. They do not yet demonstrate a reproducible signature that distinguishes an unrecognized illusion from motion sickness, workload, arousal, or the physiological effects of the maneuver itself.

For this reason, a viable model should not infer spatial disorientation from physiology alone. Electrocardiography, beat-to-beat arterial pressure where feasible, photoplethysmography, respiration, electrodermal activity, skin temperature, eye and movements should be synchronized with aircraft and head kinematics, control inputs, the visual scene, maneuver type and phase, elapsed exposure, and an individualized baseline. This context is essential because photoplethysmography is vulnerable to motion artifact and peripheral vasoconstriction, while electrodermal, cardiac, and respiratory signals are also shaped by workload, temperature, emotional arousal, and anti-G straining (Tamura et al., 2018; Rouser et al., 2026). The appropriate computational question is therefore conditional: given this maneuver, sensory environment, and pilot, what physiological response is expected? Deviations from that expectation may identify states associated with unreliable self-motion estimation but should not be labeled as an illusion until validated against an independent perceptual criterion.

A useful parallel comes from clinical vestibular disorders, where chronic spatial disorientation is evaluated through convergent measures rather than a single physiological marker (Staab et al., 2017; Strupp et al., 2017; Strupp et al., 2023). In bilateral vestibulopathy, diagnosis relies on history together with objective vestibulo-ocular reflex deficits, caloric irrigation, or rotational-chair testing, with dynamic visual acuity, posturography/Romberg testing, and vestibular-evoked myogenic potentials providing complementary information (Strupp et al., 2017; Strupp et al., 2023). In Persistent Postural-Perceptual Dizziness, symptoms are exacerbated by upright posture, active or passive motion, and complex visual environments, and visual-motion sensitivity can be quantified experimentally (Brandt et al., 2005; Pavlou et al., 2006; Kremmyda et al., 2016; Staab et al., 2017). These patient cohorts therefore support the same general principle proposed here for aviation: multimodal monitoring is most valuable when it phenotypes sensory reweighting and unstable orientation estimates, not when it is treated as a disease-like biomarker of spatial disorientation (Brandt et al., 2005; Kremmyda et al., 2016).

Obtaining that criterion requires a staged experimental program. Initial studies should compare actual tilt with translational or centrifuge stimulation while manipulating visual orientation cues. Perception should be measured continuously using a horizon-setting or forced-choice task with confidence ratings, supplemented by eye-movements and post-trial reports. These experiments can identify informative signals, quantify within- and between-pilot variability, and determine whether physiology adds predictive value beyond stimulus kinematics and behavior. Models should then be tested in increasingly realistic simulators and, subsequently, in flight in a non-interventional shadow mode. Validation should be prospective and include leave-one-pilot- and leave-one-maneuver-out tests, signal-quality gates, sensitivity and specificity, false alarms per flight hour, missed events, and time from detection to operational hazard. Importantly, a 500 ms reflex latency is not equivalent to a 500 ms detection time: sensor acquisition, artifact rejection, classification, display, and pilot response must all be included in the operational time. Real-time countermeasures become credible only if this staged process demonstrates reliable incremental prediction with sufficient lead time. Galvanic vestibular stimulation may be useful as a controlled perturbation during development, but its role as a mitigation strategy requires separate evidence of benefit and safety.

## 5 Conclusion

Spatial disorientation is best understood as a predictable failure mode of self-motion estimation arising from the physical ambiguity of otolith signals and the limits of the internal models used to interpret them. The vestibular system also contributes to rapid autonomic and respiratory regulation, and physiological changes have been measured during several experimentally induced vestibular illusions. This evidence supports multimodal physiological monitoring as a research strategy, but not yet as a direct decoder of the pilot’s percept. The next step is therefore a staged program that combines controlled perceptual labeling, individualized physiological phenotyping, and prospective validation in simulators and flight. If multimodal models can provide information beyond maneuver dynamics, workload, and behavior, they may eventually support earlier recognition of unreliable self-motion estimation and carefully evaluated adaptive countermeasures. The immediate contribution is more modest but testable: transforming spatial disorientation from a retrospective accident label into a measurable neuroergonomic process.
